# Aetiology and Outcomes of Suspected Infections of the Central Nervous System in Children in Mbarara, Uganda

**DOI:** 10.1038/s41598-017-02741-w

**Published:** 2017-06-02

**Authors:** Anne-Laure Page, Yap Boum II, Elizabeth Kemigisha, Nicolas Salez, Deborah Nanjebe, Céline Langendorf, Said Aberrane, Dan Nyehangane, Fabienne Nackers, Emmanuel Baron, Rémi Charrel, Juliet Mwanga-Amumpaire

**Affiliations:** 1 Epicentre, Paris, France; 2 Epicentre Mbarara Research Centre, Mbarara, Ouganda Uganda; 3 Mbarara University of Science and Technology, Mbarara, Ouganda Uganda; 4 Microbiology Laboratory, Créteil Hospital, Créteil, France; 5 UMR “Emergence des Pathologies Virales” (EPV: Aix-Marseille Univ - IRD 190 - Inserm 1207 - EHESP), Marseille, France; 6 Fondation IHU Mediterranee Infection, APHM Public Hospitals of Marseille, 13385 Marseille, France

## Abstract

Infections of the central nervous system (CNS) are severe conditions, leading to neurological sequelae or death. Knowledge of the causative agents is essential to develop guidelines for case management in resource-limited settings. Between August 2009 and October 2012, we conducted a prospective descriptive study of the aetiology of suspected CNS infections in children two months to 12 years old, with fever and at least one sign of CNS involvement in Mbarara Hospital, Uganda. Children were clinically evaluated on admission and discharge, and followed-up for 6 months for neurological sequelae. Pathogens were identified from cerebrospinal fluid (CSF) and blood using microbiological and molecular methods. We enrolled 459 children. *Plasmodium falciparum* (36.2%) and bacteria in CSF (13.3%) or blood (3.3%) were the most detected pathogens. Viruses were found in 27 (5.9%) children. No pathogen was isolated in 207 (45.1%) children. Patterns varied by age and HIV status. Eighty-three (18.1%) children died during hospitalisation, and 23 (5.0%) during follow-up. Forty-one (13.5%) children had neurological sequelae at the last visit. While malaria remains the main aetiology in children with suspected CNS infections, no pathogen was isolated in many children. The high mortality and high rate of neurological sequelae highlight the need for efficient diagnosis.

## Introduction

Infections of the central nervous system (CNS) in children can lead to death or neurological sequelae. Because of limited diagnostic capacities in Sub-Saharan Africa, little is known about aetiology of CNS infection in this region^1^. In addition, a variety of diseases that are not per se CNS infections, such as cerebral malaria or systemic infections, may have a clinical presentation compatible with CNS infection, further complicating the assessment of patients with suspected CNS infections. A significant proportion of children who fulfil the WHO clinical definition of cerebral malaria may have viral encephalitis or bacterial meningitis, emphasizing the need for a thorough microbiological diagnosis in patients with suspected CNS infection^2, 3^. Further, decreases in malaria across the continent and the introduction of vaccines against *Haemophilus influenzae* type B (Hib) and *Streptococcus pneumoniae* are leading to changes in the proportional burden of these pathogens^4, 5^.

Studies investigating a comprehensive range of pathogens associated with clinical picture of CNS infection are essential to adapt treatment guidelines and prevention strategies. We conducted a prospective study to investigate the infectious aetiology and outcomes in children with suspected CNS infections hospitalized in a referral hospital of a rural area of Uganda.

## Methods

### Ethical issues

The protocol was approved by the Mbarara University Institutional Ethics Committee, the Uganda National Council for Science and Technology (approval numbers MUIRC 1/& and HS 584, respectively) and the Comité de Protection des Personnes (CPP) Ile de France XI, Saint-Germain en Laye, France (reference 09013). The study was conducted following the Declaration of Helsinki. Written informed consent was obtained from parents or legal guardians of all children included in the study.

### Study setting

The study took place in the Mbarara Regional Referral Hospital (MRRH), the reference academic hospital for tertiary care in this region, and Holy Innocents Children’s Hospital (HICH), a pediatric general hospital, located in Mbarara Municipality, Mbarara District, Uganda. Mbarara Municipality is a town of 195,013 persons (2014 Population Census Provisional Results) located 300 km south-west of Kampala. Mbarara District has a population of approximately 474,144 inhabitants, and is mainly rural. In Uganda, Hib vaccination was introduced in 2002 and the pneumococcal conjugate vaccine was introduced in 2013, after the study period. The prevalence of malaria in Mbarara district ranges between 23% in rural areas and 3% in urban areas during the rainy season^6^.

### Study population

Children aged two months to 12 years admitted at the paediatric wards of MRRH and HICH were eligible for inclusion if they presented with history of fever in the past 48 hours; and with a recent onset of at least one abnormal CNS sign (inclusion criteria) described in Table 1; and if the parent or legal guardian gave informed consent. Patients were considered to have possible nosocomial infections if 48 hours after admission they developed abnormal CNS clinical signs or if they had been hospitalized for more than 48 hours during the past week. The characteristics and pathogen distribution observed in these patients were analyzed separately. Patients were included in the main analysis if no difference was found.

**Table 1:** Signs of CNS involvement (inclusion criteria).

Inclusion criteria	Definition/comment
Non traumatic reduced level of consciousness	Blantyre coma score <4 for children <9 months, Blantyre coma score <5 for older children (>9 months, preverbal) or Glasgow coma score <15 for verbal children
Prostration	Inability to sit unassisted if aged >9 months or to breastfeed if <9 months
Hypotonia/hypertonia	
Irritability	
Severe headache	Severe enough to require hospitalization
Photophobia	
Neck stiffness or bulging fontanel	
Prolonged, partial or multiple seizure(s)	On admission or history of seizures
Focal neurological signs	
Kernig sign	In children older than 18 months
Brudzinski sign	In children older than 18 months
Purpura	
Cheyne Stokes breathing	

### Inclusion and clinical management

Information on clinical exam and history were recorded in a case report form on admission and at discharge. A malaria rapid diagnostic test (SD Bioline Malaria Ag P.f/Pan, Standard Diagnostics, Gyeonggi-do, Republic of Korea) was performed as point of care diagnosis for malaria at admission. Clinicians conducted follow-up consultations at one month and six months after discharge. Children who did not return for the 1-month visit were followed up at home. At the 6-month follow-up, only those with neurological sequelae at the previous assessment were followed up at home.

Patients were treated by the attending ward clinician according to the Uganda Clinical Guidelines and protocols of the paediatric wards of the MRRH and HICH. Data quality was ensured through systematic review of all records by an internal study monitor.

### Specimen collection

Blood and CSF were collected at inclusion. Lumbar puncture, performed by a study clinician, was delayed or not performed if a contra-indication existed, including signs of raised intracranial pressure, focal neurological signs, local infection in the area of puncture, signs of bleeding disorders and cardio-respiratory compromise^7^. Two blood samples for culture were collected within 20–30 minutes of each other.

### Laboratory methods

#### Bacteriology

Bacterial culture was performed from CSF and blood. CSF was used to perform a direct Gram staining and inoculated onto chocolate agar, blood agar and Schaedler broth. One milliliter of each blood sample was inoculated into a blood culture bottle (SIGNAL Blood culture System, OXOID, UK). A blood culture was considered negative after seven days of incubation or negative subculture. Gram staining results from positive blood cultures determined subsequent choice of enriched and/or selective media for subculture. Colonies were identified using standard biochemical methods.

Mycobacterial culture was initially performed systematically on CSF, or, starting in December 2010 (after patient 189), only on patients with a score of 6 or higher using the case definition for tuberculous meningitis for clinical research proposed by Marais *et al*.^8^. A recommended volume of 500 µL of CSF before December 2010, or 500 µL to 1 ml thereafter, was inoculated on Mycobacterial Growth Indicator Tubes (MGIT, BD Diagnostics, Sparks, USA) and cultured according to standard procedures^9^.

#### Polymerase chain reaction (PCR)

PCR was performed at the Epicentre laboratory in Mbarara, until November 2010 (patient 183) and in Marseille, France, thereafter. DNA and RNA were extracted from the CSF samples using the EZ1 system (Qiagen GmbH, Hilden, Germany) and EZ1 Virus Mini Kit v2.0 kits. Real-time PCR for the following agents was performed systematically: *S. pneumoniae*, *H. influenzae* type B, *Salmonella*, *Listeria monocytogenes*, enterovirus, herpes simplex virus 1/2 (HSV-1/2), varicella zoster virus (VZV), mumps, measles, human herpesvirus 6 (HHV6), pan-flavivirus, cytomegalovirus (CMV), adenovirus and rabies^10–20^. In addition, the first 384 patients (out of 480 patients included) were tested for *N. meningitidis* and *M. tuberculosis*, which were then discontinued since all results were negative. All the PCRs, whether performed in Mbarara or Marseille, were done using the same reagents, including positive controls, prepared in Marseille. For the PCR performed in Mbarara, a quality control was done in Marseille on a sub-sample of specimens. These methods and reagents are used in routine in Marseille in the hospital diagnostic laboratory, with appropriate controls, and have been validated by participation to QCMD proficiency testing with results ranging from good to excellent, particularly for sensitivity with low viral load specimens.

#### Other laboratory investigations

From each child, thin and thick blood films were prepared, Giemsa-stained, and read by experienced microscopists for detection of *Plasmodium* spp. Cryptococcal infections were investigated using the Cryptococcal Antigen Latex Agglutination System (CALAS®, Meridian Bioscience, Inc., Cincinnati, USA) followed, if positive, by examination of India ink-stained smear. After counselling and informed consent, HIV serology was performed using a serial algorithm consisting of Determine HIV 1/2 (Alere, USA), followed by HIV 1/2 STAT-PAK (Chembio, USA) for reactive samples, and Uni-Gold HIV (Trinity Biotech, Ireland) as a tie-breaker for discordant results, according to national guidelines.

Serology was used whenever possible to confirm HHV-6 and mumps (Anti-Parotitis Virus/IgG – IgM, Siemens) infection detected by PCR. The serology was considered positive if IgM were detected in the serum at inclusion or discharge.

Additional biological analyses, including CSF cell counts, hematology and biochemistry were performed and will be presented elsewhere.

#### Definitions

Malaria was considered the cause of infection if trophozoites were detected by smear microscopy or, in cases where a child had been on antimalarial drugs, the rapid test was positive regardless of blood smear result. Malaria was subclassified as cerebral malaria if the child had impaired level of consciousness (coma defined as Glasgow score <11 or Blantyre score <3) in the absence of other causes of altered consciousness^21^. Bacterial meningitis was considered if a bacterial pathogen was detected in the CSF by culture, PCR or Gram staining. Viral infection was considered when a virus was detected by PCR in the CSF, regardless of serology results.

Neurological sequelae were defined as the occurrence or persistence of an abnormal neurological finding that the child did not have before this acute illness. Sequalae were classified using the Medical Dictionary for Regulatory Activities (MedDRA, www.meddra.org).

#### Statistical analysis

Data analysis was done using Stata® (version 12, College Station, Texas, USA). For categorical variables, we compared proportions using the Pearson chi-square or Fisher exact tests. Crude odds ratios (ORs) were estimated with 95% confidence intervals (CI). Two multivariate logistic regression models were built, both including age, sex, and all variables associated with a higher risk of death in the univariate analysis (p < 0.05), and one also including the laboratory-confirmed diagnosis.

## Results

### Population description

**Figure 1:**
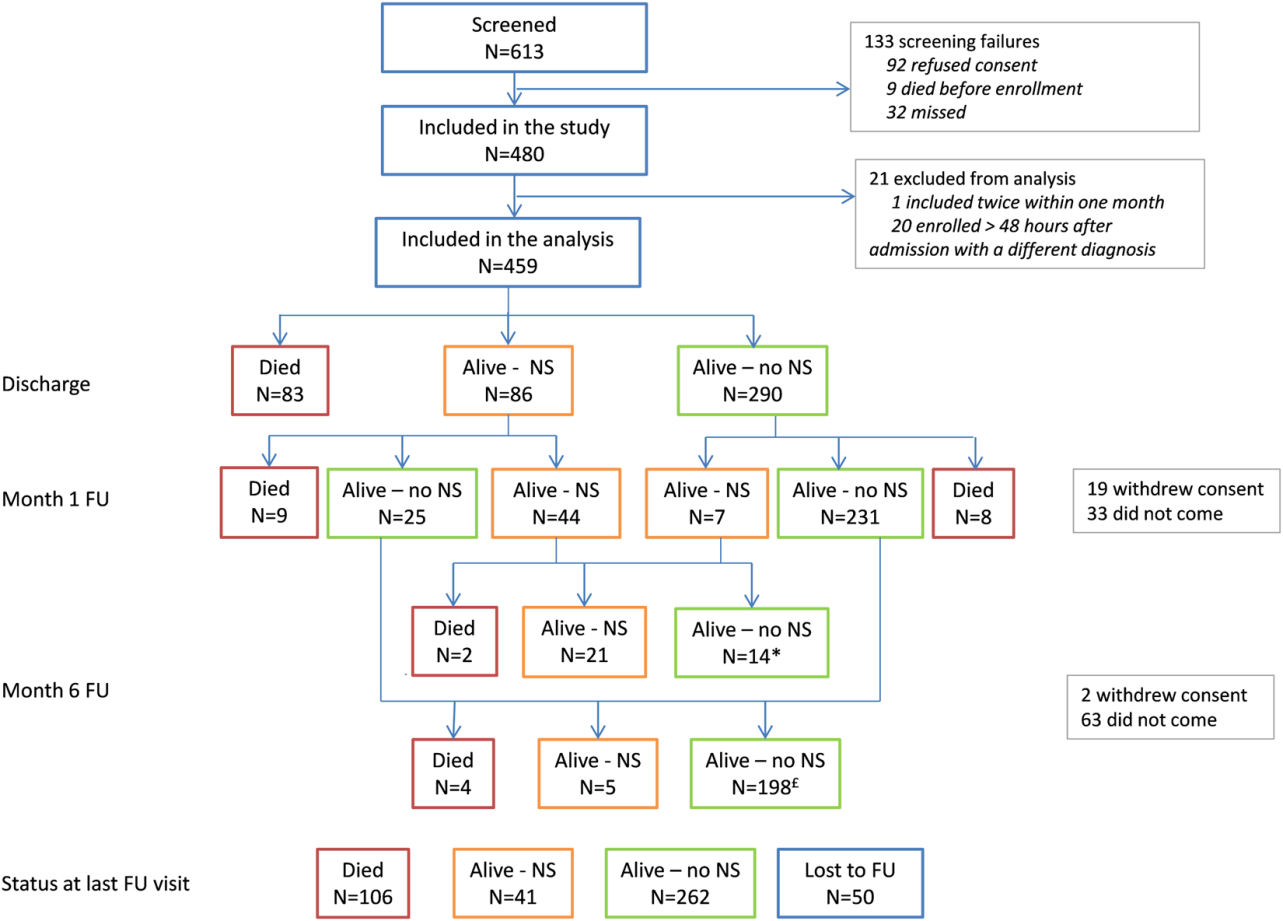
Study flow-chart. NS: Neurological sequelae; FU: follow-up; Lost to FU: withdrew consent or did not come to any FU visit * including 1 with neurological sequelae at discharge and missing visit at month 1 £ including 1 with no neurological sequelae at discharge and missing visit at month 1.

Between August 2009 and October 2012, we screened 613 children and included 480 in the study. Among them, 13 were hospitalized elsewhere for at least two days before admission, and 20 developed clinical signs of CNS involvement more than 48 h after admission. The profile of children hospitalized elsewhere for more than 2 days prior to admission at MRRH was globally similar to other children in terms of general characteristics and bacteria found (eTable 1). These children were thus included in the main analysis presented here. In contrast, the higher proportion of bacteremia and different type of bacteria (*K. pneumoniae*, *Brevibacterium* spp., coagulase negative *Staphylococcus*) in the group with delayed inclusion and different diagnoses on admission and inclusion suggests that some of these might be nosocomial infections. To be noted also that the two *K. pneumoniae* isolated from children in this group showed an extended-spectrum beta-lactamase. These children were thus excluded from the main analysis as well as one child included twice in the study within one month. Finally, 459 children were included in the present analysis (Fig. 1). Of these, 291 (63.4%) were boys and the median age was 30 months (interquartile range (IQR): 11–60) (Table 2).

**Table 2:** Demographic and clinical characteristics on inclusion and their association with the risk of dying during hospitalization, univariate and multivariate analyses (including or not laboratory-confirmed diagnoses).

Characteristics	Total (N = 459)	Died	Odds ratio (95% CI)		
	n (% of all)	n (% of category)	Univariate	Multivariate	Multivariate - with diagnosis
***Patients characteristics***					
Sex					
Male	291 (63.4)	58 (19.9)	Ref		Ref
Female	168 (36.6)	25 (14.9)	0.70 (0.42–1.17)	0.69 (0.40–1.21)	0.66 (0.37–1.17)
Age (months)					
2–11	122 (26.6)	27 (22.1)	1.21 (0.64–2.28)	0.97 (0.48–1.98)	0.94 (0.45–1.97)
12–59	221 (48.2)	34 (15.4)	0.78 (0.43–1.40)	0.67 (0.35–1.28)	0.74 (0.38–1.45)
≥60	116 (25.3)	22 (19.0)	Ref	Ref	Ref
HIV serology (N = 451)					
Positive*	44 (9.8)	16 (36.4)	3.01 (1.53–5.87)	2.78 (1.30–5.93)	2.50 (1.12–5.55)
Negative	407 (90.2)	65 (16.0)	Ref	Ref	Ref
Malnutrition^a^ < 5 years (N = 311)					
No malnutrition	242 (77.8)	37 (15.3)	Ref		
Moderate	36 (11.6)	8 (22.2)	1.58 (0.67–3.74)		
Severe	33 (10.6)	6 (18.2)	1.23 (0.48–3.19)		
***Inclusion criteria*** ^**b**^					
Reduced consciousness* ^**,c**^	329 (71.7)	74 (22.5)	3.90 (1.89–8.06)	5.18 (1.71–15.7)	5.29 (1.68–16.7)
Coma* ^**,c**^	226 (49.2)	58 (25.7)	2.87 (1.72–4.79)		
History of seizures	299 (65.1)	59 (19.7)	1.39 (0.83–2.34)		
Seizures	238 (51.9)	46 (19.3)	1.19 (0.74–1.92)		
Hypotonia	69 (15.0)	16 (23.2)	1.46 (0.78–2.70)		
Hypertonia	95 (20.7)	19 (20.0)	1.17 (0.66–2.07)		
Neck stiffness*	100 (21.8)	25 (25.0)	1.73 (1.01–2.95)	1.54 (0.82–2.89)	1.53 (0.78–2.99)
Prostration*	63 (13.7)	5 (7.9)	0.35 (0.14–0.91)	1.19 (0.28–4.98)	1.33 (0.30–5.82)
Bulging fontanel	29 (6.3)	6 (20.7)	1.20 (0.47–3.04)		
Kernig or Brudzinski sign	27 (5.9)	8 (29.6)	2.0 (0.84–4.75)		
Irritability	135 (29.4)	17 (12.6)	0.56 (0.31–1.00)		
Headache	52 (11.3)	9 (17.3)	0.94 (0.44–2.01)		
Photophobia	13 (2.8)	4 (30.8)	2.06 (0.62–6.87)		
Focal neurological sign	19 (4.1)	5 (26.3)	1.66 (0.58–4.74)		
Cheyne Stokes breathing	16 (3.5)	4 (25.0)	1.54 (0.48–4.89)		
Purpura	3 (0.7)	0 (0)			
***Clinical signs at inclusion***					
Diarrhea	85 (18.5)	19 (22.4)	1.39 (0.78–2.48)		
Vomitting	126 (27.5)	29 (23.0)	1.54 (0.93–2.56)		
Dehydration*	80 (17.4)	22 (27.5)	1.98 (1.13–3.47)	1.16 (0.60–2.24)	1.26 (0.64–2.49)
Hepatomegaly	181 (39.4)	39 (21.6)	1.46 (0.90–2.36)		
Splenomegaly	130 (28.3)	22 (16.9)	0.89 (0.52–1.53)		
Respiratory distress*	136 (29.6)	42 (30.9)	3.07 (1.88–5.01)	2.41 (1.36–4.25)	2.28 (1.27–4.10)
Abnormal lung auscultation*	63 (13.7)	20 (31.8)	2.46 (1.35–4.46)	1.14 (0.57–2.27)	1.10 (0.53–2.28)
Decompensated anemia	48 (10.5)	11 (22.9)	1.40 (0.68–2.87)		
Jaundice	55 (12.0)	15 (27.3)	1.85 (0.97–3.54)		
Delayed capillary refill*	17 (3.7)	7 (41.2)	3.37 (1.24–9.14)	1.74 (0.48–6.25)	2.77 (0.68–11.2)
Cold extremities*	20 (4.3)	8 (40.0)	3.24 (1.28–8.19)	1.40 (0.40–4.90)	1.58 (0.42–5.93)
Cyanosis	4 (0.9)	2 (50.0)	4.62 (0.64–33.3)		
***Laboratory-confirmed diagnosis***					
Malaria	46 (10.0)	1 (2.2)	0.10 (0.01–0.73)		0.12 (0.01–0.92)
Cerebral malaria	109 (23.8)	21 (19.3)	1.06 (0.59–1.92)		0.92 (0.47–1.80)
Bacterial meningitis	47 (10.2)	12 (25.5)	1.52 (0.72–3.21)		1.50 (0.65–3.49)
Bacteremia	11 (2.4)	2 (18.2)	0.99 (0.21–4.76)		1.31 (0.2–7.17)
Viral infection	9 (2.0)	4 (44.4)	3.56 (0.91–13.9)		3.92 (0.82–18.8)
Cryptococcal infection	2 (0.4)	1 (50.0)	4.45 (0.27–72.7)		4.02 (0.12–139.0)
Tuberculous meningitis	4 (0.9)	1 (25.0)	1.48 (0.15–14.6)		1.57 (0.14–18.1)
Mixed malaria - bacterial infection^d^	6 (1.3)	1 (16.7)	0.89 (0.10–7.83)		0.81 (0.08–8.63)
Mixed viral-bacterial infection^e^	12 (2.6)	2 (16.7)	0.89 (0.19–4.23)		0.55 (0.10–3.02)
Mixed viral-other infection	6 (1.3)	0 (0)	—		—
No lab-confirmed diagnosis	207 (45.1)	38 (18.4)	Ref		Ref

95% CI: 95% confidence interval;

*Variables associated with a higher risk of death in univariate analysis and included in the multivariate analysis.

^a^Moderate malnutrition was defined as a weight for height z-score between −2 and −3 and severe malnutrition was defined as a weight for height z-score < −3 and/or bipedal edema;

^b^Criteria reported as “not applicable” (due to age groups) or “don’t know” (eg. headache in infants or unconscious child) were considered as negative.

^c^Reduced consciousness was defined as a Blantyre coma score < 4 in children < 9 months, Blantyre coma score < 5 in preverbal children >9 months, or Glasgow coma score < 15 in verbal children. Coma was defined as Blantyre coma score < 3 or Glasgow coma score < 11.

^d^Including malaria and bacterial meningitis (n = 3) or bacteraemia (n = 3).

^e^Including virus and bacterial meningitis (n = 11) or bacteraemia (n = 1).

Of 451 patients for whom representatives accepted HIV testing, 44 (9.8%) were positive by serology. Among 297 children over 18 months of age, 21 (7.1%) were HIV-infected. The final status of the 23/154 (14.9%) children less than 18 months with perinatal HIV exposure was not assessed as part of this study. Among children over four months of age, 343/416 (82.4%) reported receiving a full course of Hib vaccine.

More than half (252/459 = 54.9%) of patients had received a treatment within seven days before admission, including antimalarials in 114 (24.8%), antibiotics in 42 (9.1%), and both antimalarials and antibiotics in 63 (13.7%). Fifty-eight (12.6%) patients were hospitalised somewhere else for at least one night before admission.

Over half of the children presented with three or four signs outlined in the inclusion criteria (n = 239, 52.1%). The most frequent inclusion criteria were reduced consciousness and active seizures on admission or history of seizures (Table 2).

### Laboratory-confirmed aetiologies

CSF was collected in 404 patients (88.0%). Reasons for no CSF collection included contra-indication (n = 33), failed lumbar puncture (n = 11), or lumbar puncture performed before inclusion in the study (n = 3). Reasons were not reported for 8 patients. Blood was collected in 456 patients (99.3%).

Malaria was the most frequent laboratory–confirmed aetiology with 166 patients (36.2%), of whom 109 (66.8%) were classified as cerebral malaria, while the others had signs of CNS involvement (Table 1) but not coma or also had another pathogen identified (Table 3). Median parasitemia was similar in those classified as cerebral malaria (20508, IQR: 1293-123019) and non-cerebral malaria (19640, IQR: 1213-179801).

**Table 3:** Laboratory-confirmed aetiology and corresponding pathogens identified.

Laboratory-confirmed aetiology	n (%)	Pathogens
***Mono-infections***	***228 (49.7)***	
Malaria	46 (10.0)	Pf (46)
Cerebral malaria	109 (23.8)	Pf (104); Pf + *P. malariae* (2); Pf + *P. ovale* (2); Pf + *P. vivax* (1)
Bacterial meningitis	47 (10.2)	*S. pneumoniae* (28), NTS (5), Hib (5), S. Typhi (1), GPC (7), GNC (1)
Bacteremia	11 (2.4)	*S. pneumoniae* (4), NTS (3), S. Typhi (2), E. coli (2)
Viral infection	9 (2.0)	HHV6 (5), VZV (2), mumps (1), CMV (1)
Cryptococcal infection	2 (0.4)	
Tuberculous meningitis	4 (0.9)	
***Mixed infections***	***24 (5.2)***	
Malaria + bacterial meningitis	3 (0.7)	*S. pneumoniae* (1), NTS (1), *Brevibacterium* (1)
Malaria + bacteraemia	3 (0.7)	*S. pneumoniae* (1), NTS (1), *S. epidermidis* (1)
Viral infection + bacterial meningitis	11 (2.4)	CMV + *S. pneumoniae* (3); CMV + Hib (2); CMV + NTS (1); CMV + GPC (1); mumps + GPC (1); enterovirus + *S. pneumoniae* (1); HHV-6 + S. pneumoniae (1); HHV-6 + GCP (1)
Viral infection + bacteremia	1 (0.2)	mumps + *S. aureus* (1);
Viral infection + other infection	6 (1.3)	HHV-6 + Pf (3); CMV + Pf (1); mumps + Pf (1); HHV-6 + *Cryptococcus* (1)
***No laboratory-confirmed diagnosis***	***207 (45.1)***	

CMV, cytomegalovirus; GNC, Gram negative cocci; GPC, Gram positive cocci; Hib, *Haemophilus influenzae* type b; NTS, Non typhoidal *Salmonella*; Pf, *Plasmodium falciparum*; VZV, Varicella zoster virus.

In total, 61 patients (13.3%) had a bacterial pathogen detected in CSF, either alone (n = 47) or in co-infection (n = 14). *S. pneumoniae* was the most frequently isolated pathogens in CSF by culture or PCR (n = 34), followed by non-typhoidal *Salmonella* (NTS, n = 8), and Hib (n = 7) (Table 3). Eleven patients had a diagnosis of bacterial meningitis based on CSF Gram staining only. In addition, 15 patients (3.3%) had bacteremia (11 alone, 4 in co-infection). Again, the most frequent pathogens were *S. pneumoniae* and NTS.

A confirmed or probable viral aetiology was found in 27 (5.9%) patients (11 HHV-6, 9 CMV, 4 mumps, 2 VZV, and 1 enterovirus). Among them, nine patients had viral infection only (5 HHV-6, 2 VZV, 1 mumps, and 1 CMV), while the virus was associated with another pathogen in 18 other patients, mostly with bacterial infections (n = 12) (Table 3).

Four patients had laboratory-confirmed tuberculous meningitis, while three had a laboratory-confirmed cryptoccocal infection, one of which was a co-infection with HHV-6.

No pathogen was isolated in the blood or in the CSF of 207 children (45.1%). Their mean number of inclusion criteria was slightly lower than those with a confirmed aetiology (3.8 versus 4.2; p = 0.02). They were also less likely to have taken antimalarials (32.2% versus 45.4%, p = 0.008), but more likely to have taken antibiotics (28.9% versus 19.6%, p = 0.07) prior to admission. Some of these children (n = 36) also had other clinically suspected systemic diagnoses other than CNS infections, such as pneumonia (7), or sepsis (9). Viral meningoencephalitis was clinically suspected in 98 (46.4%) of them.

### Aetiology by age group and HIV status

**Figure 2:**
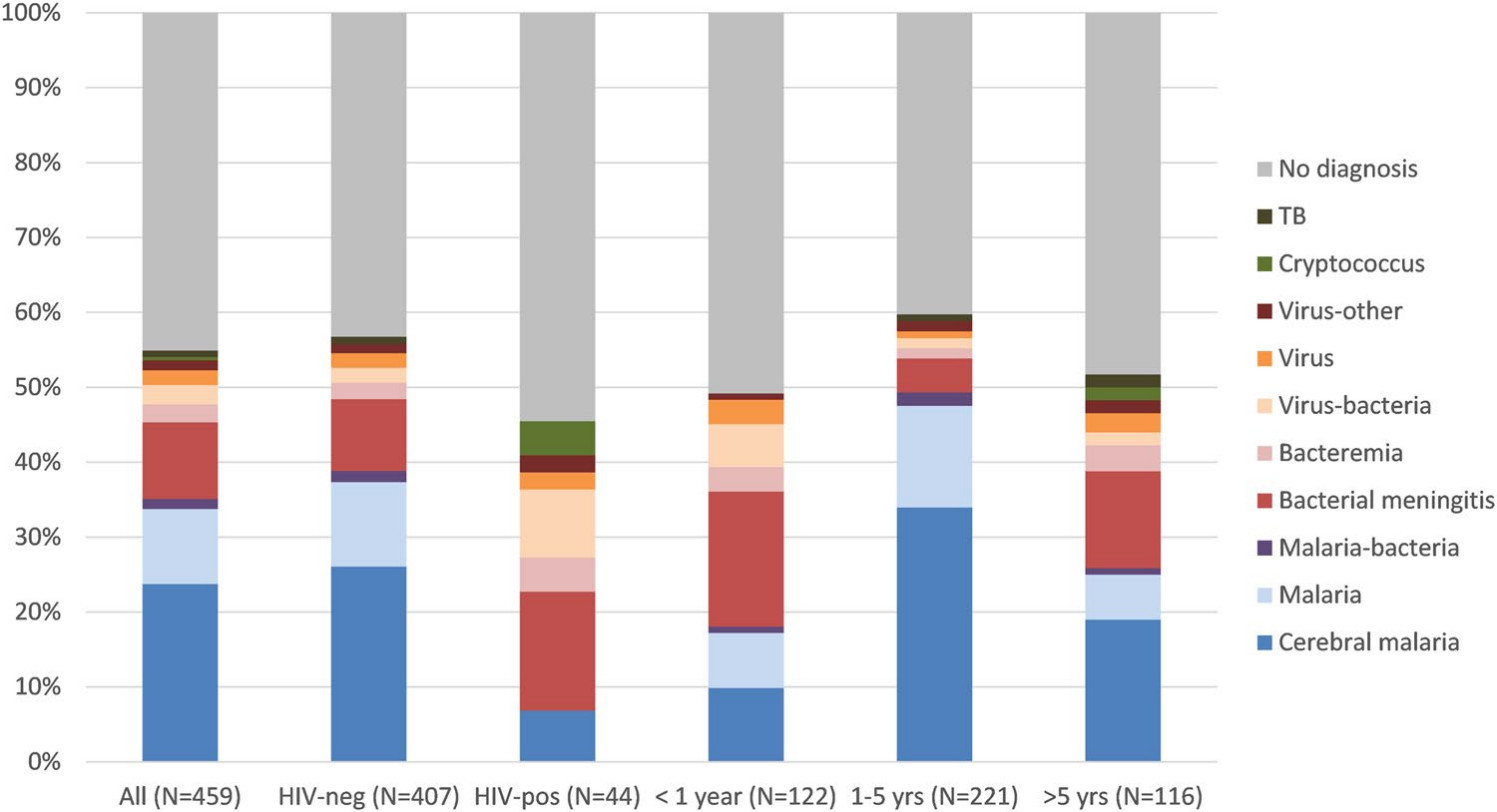
Laboratory-confirmed etiology of CNS infections by HIV status and age group: all children (A, N = 459), HIV-positive (B, N = 44) HIV-negative (C, N = 415), age below one year (D, N = 122), one to 5 years (E, N = 221) and above 5 years (F, N = 116). HIV-positive include all patients positive for HIV by serology, including those less than 18 months of age (HIV-exposed).

Bacterial meninigitis was more frequent among children less than one year (18.0%) or older than five years (12.9%) compared to children aged 1–5 years (4.5%, p < 0.001), who suffered more from cerebral malaria (33.9% versus 9.8% in < 1 year-old and 19.0% in >5 year-old, p < 0.001, Fig. 2). All seven cases of Hib meningitis occurred in children less than one year, mostly unvaccinated (n = 5/7).

Bacterial meningitis was also more frequent in HIV-positive children, including those with perinatal HIV exposure, though the difference was not statistically significant (15.9% versus 9.6%; p = 0.19), while cerebral malaria was more frequent among HIV-negative patients (25.5% versus 6.8%; p = 0.004) (Fig. 2). All cases of cryptococcal meningitis were in HIV-positive children older than five years, while all tuberculous meningitis occurred in HIV-negative children.

### Outcomes

**Figure 3:**
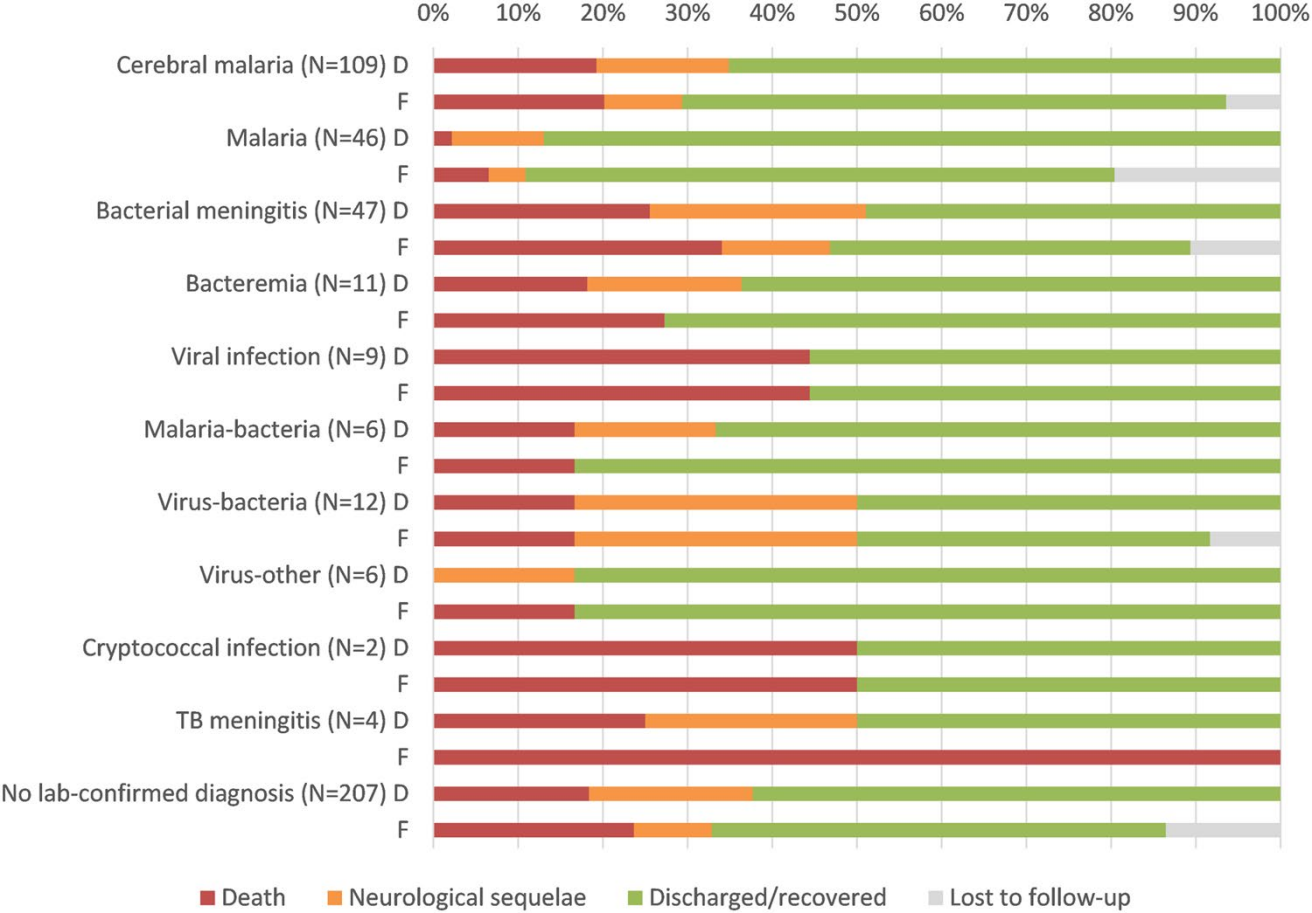
Outcomes at discharge and at the last follow-up visit by etiology. D: outcome at discharge, F: outcome at the last follow up visit.

Eighty three (18.1%) children died at the hospital and another 23 (5.0%) children during follow-up, leading to a total of 106 deaths (23.1%) (Fig. 1). In addition, 86 (22.9%) of the children alive at discharge presented neurological sequelae, and 41 (13.5% of survivors excluding lost to follow-up) had neurological sequelae at their last follow up visit. The outcomes at discharge and at the last follow-up visit by type of infection are presented in Fig. 3.

In the multivariate analysis, children with a positive HIV serology, reduced consciousness, or respiratory distress on inclusion were more at risk of dying during hospitalisation, while children with non-cerebral malaria were at reduced risk of dying at the hospital compared to other aetiologies (Table 2). All four children with tuberculous meningitis had died by the end of the 6-month follow-up (Fig. 3). Around one third of children with neurological sequelae had more than one impairment (38.6% at discharge and 29.3% at last follow up visit). The most frequent sequelae were motor dysfunction (Table 4).

**Table 4:** Neurological sequelae identified at discharge and at the last follow-up visit among all, bacterial meningitis and cerebral malaria cases.

	Discharge	Last follow-up visit			
	Total (N = 86)	Total (N = 41)	Bacterial meningitis (N = 11)	Cerebral malaria (N = 10)	No diagnosis (N = 18)
Motor dysfunction^a^	67 (77.9)	30 (73.2)	9 (81.8)	8 (80.0)	13 (72.2)
Seizures	19 (22.1)	6 (14.6)	0 (0)	3 (30.0)	2 (11.1)
Blindness	17 (19.8)	5 (12.2)	4 (36.4)	0 (0)	1 (5.6)
Hearing loss	11 (12.8)	4 (9.8)	4 (36.4)	0 (0)	0 (0)
Cranial nerve palsies	15 (17.4)	4 (9.8)	1 (9.1)	2 (20.0)	1 (5.6)
Neuropsychiatric disorders	7 (8.1)	5 (12.2)	0 (0)	1 (10.0)	2 (11.1)
Speech impairment	5 (5.8)	1 (2.4)	0 (0)	1 (10.0)	0 (0)
Cognitive disorder	0 (0)	2 (4.9)	2 (18.2)	0 (0)	0 (0)
Other^b^	2 (2.3)	1 (2.4)	0 (0)	0 (0)	1 (5.6)

^a^Including hemiplegia/paresia, quadriplegia/paresia, extrapyramidal rigidity, hypotonia, hypertonia, and cerebellar ataxia.

^b^Including foot drop, regressed development.

## Discussion

This study with comprehensive laboratory investigations allowed us to identify an infectious aetiology in slightly more than half of hospitalised children with a suspected infection of the CNS. Despite a decrease in malaria prevalence in the area during the study^6^, cerebral malaria was the main aetiology in our cohort, followed by bacterial meningitis. Although we investigated a large range of viruses, we found few viral infections, most of which were in children co-infected with another pathogen.

Cerebral malaria particularly affected children 1–5 years of age, with a prevalence of about 35% in this age group in line with other reports from the region^22^, despite the dramatic decrease in malaria incidence^5^. In contrast to other recent studies^23, 24^, cerebral malaria was more common among HIV negative children. Although not recorded, HIV positive and exposed children in this study would likely have received cotrimoxazole prophylaxis, which is also effective in malaria prevention^25^.

Bacterial meningitis was the second most common aetiology with 13.3% of cases overall, including mixed infections. This is slightly higher than reported in the few studies on the aetiology of CNS infections or reduced consciousness outside of the meningitis belt^22, 26^. This is likely due to the use of PCR, which allowed the identification of bacteria in the CSF in 22 cases in addition to the 28 detected by culture and 11 by direct examination. As reported in other African countries^27–29^, *S. pneumoniae* was the main agent of bacterial meningitis, representing about two thirds of all confirmed cases. Despite reported good coverage of Hib vaccine in Uganda^4^, Hib was found in around 10% of confirmed bacterial meningitis, but only in children less than one year of age and mostly unvaccinated. NTS, the second cause of bacterial meningitis in our cohort, is becoming increasingly recognized as an important cause of bacterial meningitis in sub-Saharan Africa^30^. It is the second or third cause in several countries after the introduction of the Hib vaccine^29, 31, 32^, and might even become the leading cause of bacterial meningitis after effective introduction of pneumococcal vaccine.

Although viral infections are often suspected in the absence of any other pathogen, we identified few viral infections (5.9%). This is in contrast with a recent study in Malawi that identified a virus in 23% of children with non-bacterial CNS infections^33^, suggesting potential geographical disparities. Interestingly, HSV-1, which was the most frequent virus isolated in a study in Kenya^34^ and was clinically suspected in 3% of the patients included in this study (who received acyclovir), was never confirmed by PCR.

Confirming the role of a pathogen as the causative agent of a specific condition is difficult, particularly when several possible pathogens are found concomitantly. In Kenya and Malawi, 10% and 27% of children with malaria parasitemia also had viral infections^33, 34^. In Malawi, children with malaria and viral infections were at increased risk of dying, suggesting that both pathogens could contribute to the disease. In our study, most viral infections were found in co-infections with bacteria or other pathogens. However, we did not find any association with increased severity, maybe due to the small numbers, and we cannot conclude whether one or the other, or both pathogens were the cause of the symptoms. More data is needed to establish causality, particularly for less-well characterized agents such as HHV-6^35^, or viruses associated with persistent infection, like herpesviruses, detection of which might reflect re-activation rather than primary disease. Out of seven patients positive for HHV-6 by PCR and for whom serology could be performed, only two showed IgM at inclusion or discharge, suggesting absence of true viral replication in the others. The proportion of viral infections reported here might actually be an over-estimate of the proportion of CNS infections caused by viruses in this setting.

Our study had several limitations. As stated above, the causal role of pathogenic agents is sometimes difficult to establish. Some cases of asymptomatic parasitemia might have been misclassified. Additional examinations such as fundoscopy or plasma PfHRP2 could have helped in attributing final diagnoses^36, 37^, and the inclusion of controls could have helped in determining attributable fraction. Despite the wide range of agents targeted by our investigations, some agents could have been missed, such as non-cultivable bacteria, parasites other than *Plasmodium* spp or viral agents not included in our PCR panel. The relatively wide inclusion criteria in the study also led to inclusion of patients with diseases with no CNS infectious component. In addition, non-infectious causes, such as metabolic disorders, were not investigated here. Finally, some subtle neurological impairments might have been missed in our study due to the relatively short follow-up period and the neurological clinical assessment done, which focussed mostly on moderate to severe neurological sequelae. In particular, we did not use a psychologist or specialized scales to assess the children’s cognitive abilities. All these factors might explain why cognitive impairments were not frequently reported here, while they represent one the main long-term sequelae associated with bacterial meningitis and cerebral malaria, after most of the gross neurological sequelae have resolved^38, 39^.

In conclusion, this study confirms the high mortality and high incidence of neurological sequelae in children presenting with a clinical picture of suspected CNS infections. Despite decreasing prevalence in Uganda and elsewhere in Africa, malaria remains the most common aetiology in children with suspected CNS infection in this setting, followed by bacterial meningitis,. The predominance of *S. pneumoniae* emphasizes the need for effective introduction of pneumococcal conjugate vaccine. Although the prevalence of tuberculous and cryptococcal meningitis were low, their high toll in terms of death and sequelae point to the need to improveguidelines for diagnosis and empiric treatment. Finally, the high proportion of patients without a laboratory-confirmed aetiology calls for more performant diagnostic techniques, pathogen discovery using metagenomics techniques, as well as post mortem studies to better understand these highly severe conditions.

## Electronic supplementary material

Supplementary Information﻿.
