# Supplementary material for: Aetiology and Outcomes of Suspected Infections of the Central Nervous System in Children in Mbarara, Uganda
Source: Sci Rep. 2017 Jun 2;7:2728. doi: 10.1038/s41598-017-02741-w (PMC5457409; doi:10.1038/s41598-017-02741-w)
Supplement: Supplementary file 1 — Supplementary Information﻿. [file 41598_2017_2741_MOESM1_ESM.pdf]

Aetiology and Outcomes of Suspected Infections of the Central Nervous System in Children in Mbarara, Uganda

Anne-Laure Page[1], PhD, Yap Boum II[2,3], PhD, Elizabeth Kemigisha[2,3], MD,MMED, Nicolas Salez[5], PhD, Deborah Nanjebe[2], MSc, Céline Langendorf[1], PharmD, Said Aberrane[4], MD, Dan Nyehangane[2], MSc, Fabienne Nackers[1], MD,PhD, Emmanuel Baron[1], MD, Rémi Charrel[5,6], MD, PhD, Juliet Mwanga-Amumpaire [2,3] *, MD, MMED

eTable 1. Characteristics of patients included two days after admission or prior hospitalization elsewhere for more than two days compared to those included within two days of admission

	Included within 2 days of admission[$] (N=447)	Included > 2 days after admission (N=20)	Hospitalised > 2 days in past week (N=13)
Socio-demographic			
Sex, % males	283 (63.3)	9 (45.0)	9 (69.2)
Age, median (IQR)	30 (11-60)	10 (6.5-60.5)	28 (8-34)
Inclusion criteria			
Reduced consciousness	125 (28.0)	12 (60.0)	6 (46.2)
Seizures on admission	235 (52.6)	5 (25.0)	4 (30.8)
History of seizures	296 (66.2)	5 (25.0)	4 (30.8)
Laboratory-confirmed diagnosis			
Malaria	45 (10.1)	1 (5.0)	1 (7.7)
Cerebral malaria	108 (24.2)	1 (5.0)	1 (7.7)
Bacterial meningitis	44 (9.8)	3* (15)	3[£] (30.8)
Bacteremia	10 (2.2)	3* (15)	1[£] (7.7)
Virus	9 (2.0)	0	0
Crypto	2 (0.5)	0	0
TB	4 (0.9)	0	0
Mixed malaria -bacteria	6 (1.3)	1* (5)	0 (0)
Mixed viral-bacteria	11 (2.5)	0 (0)	1[£] (7.7)
Mixed viral-other	5 (1.1)	0	0
No laboratory diagnosis	202 (45.2)	11 (55.0)	6 (46.2)
Death	82 (18.4)	5 (25.0)	1 (7.8)

$ Includes one patient included twice within a month, later excluded

* *S. pneumoniae* (2), *K. pneumoniae* (2), Hib (1), coagulase-negative *Staphylococcus* (1), *Brevibacterium* spp. (1)

£ Hib (2), *Salmonella* spp. (2), *S. pneumoniae* (1)
